# Hastening Slowly

**Published:** 1890-05

**Authors:** 


### HASTENING SLOWLY.

Dr. T. C. Osborn, of Cleburne, an honorary member of the Texas State Medical Association, and formerly a member of the Alabama Medical Association, the best organized and consequently the strongest and most influential medical body in the United States, introduced a resolution at the recent meeting, at Fort Worth, providing for dividing the State into four districts, and electing a Vice-President for each district, with power to organize the profession, and other functions. It was a move in the right direction, and had it been promptly adopted, instead of postponed, it would have marked a new era in the Association's career, and given it a new impetus. Dr. Osborn is familiar with the machinery of the Alabama Society, whereby it has not only placed itself in the foremost rank of medical organizations, but has succeeded in making itself really the medical department of the State government. Its constitution is a part of the organic law of the State. The Association has entire charge of the matter of regulating the practice, of appointing all medical officers and boards of health, etc., of registering vital statistics, and of licensing physicians to practice. This is the desideratum for which the JOURNAL has striven for five years; but every request made of our legislature has been refused. Why? Because the profession is not organized,—do not *pull together*. It will be remembered that the bill to regulate the practice, which was presented by a special commitree, in '85, after having been read before and approved by the State Medical Association, was defeated by a part of the profession who were not in accord with the Association. Many of those who opposed it are now zealous members, and have put their shoulders to the wheel to help push along the work of regeneration.

The State is overrun with quacks, and they are doing an incalculable amount of harm. It devolves on the medical profession to remedy this evil, and it can never be done until the profession is a unit, and can speak to the legislature "as one having authority."

Dr. Osborn's excellent suggestion should have been promptly adopted and put into operation; it would have facilitated matters very greatly. He is one of the Elders, and his voice should receive the most respectful consideration. For our own part, we were much chagrined that the resolution went over till next meeting.

